# Clinical and Operational Effects of Emergency Department Crowding: A Systematic Review

**DOI:** 10.7759/cureus.100560

**Published:** 2026-01-01

**Authors:** Anas E Ahmed, Abdulrahman A Aqeel, Mohammed Y Ghazwani, Abdulmohsen N Alenzi, Faisal M Alruwaili, Abdulmajeed S Alsharari, Yaseer H Alharbi, Albatool A Aldahaas, Mozoon M AlMajed, Faris A Alshahrani

**Affiliations:** 1 Community Medicine, Jazan University, Jazan, SAU; 2 Emergency Medicine, Prince Mohammed bin Nasser Hospital, Jazan, SAU; 3 Medicine, Jazan University, Jazan, SAU; 4 Medicine, Al-Jouf University, Sakakah, SAU; 5 Medicine, Hail University, Hail, SAU; 6 Medicine, King Abdulaziz University, Jeddah, SAU; 7 Medicine, King Faisal University, AlHassa, SAU; 8 Medicine, Tabuk University, Tabuk, SAU

**Keywords:** boarding, care delays, clinical processes, emergency care, emergency department crowding, healthcare system performance, length of stay, operational efficiency, patient outcomes, systematic review

## Abstract

Emergency department (ED) crowding, defined as a mismatch between ED capacity and patient care demands, remains a global challenge linked to delays in care, reduced quality of services, and increased risk of adverse outcomes. This review synthesized thirteen observational studies evaluating the effects of ED crowding on patient outcomes, care processes, and healthcare system performance, including mortality, delays in assessment and treatment, diagnostic and therapeutic timeliness, boarding duration, ED and inpatient length of stay, readmissions, errors, and staff-related impacts. Findings showed that ED crowding consistently resulted in delayed clinical processes, particularly analgesia administration, antibiotic initiation, stroke evaluation, trauma care, and sepsis management, alongside increased ED and inpatient length of stay, higher rates of hospital-acquired complications, diagnostic delays, and reduced adherence to evidence-based practices. Reports on mortality were inconsistent, with some studies identifying an association and others finding no independent effect after adjusting for illness severity, reflecting variability in study design and measurement. Overall, ED crowding was associated with operational strain, increased admission rates, and heightened resource utilization. These findings indicate that ED crowding adversely affects the timeliness and quality of emergency care and contributes to broader system inefficiencies, underscoring the need for coordinated strategies focused on improving patient flow, increasing bed capacity, and reducing boarding to mitigate its impact.

## Introduction and background

Emergency departments (EDs) are the frontline of acute care, providing time-critical evaluation and treatment for a wide range of medical, surgical, and traumatic emergencies [1-3]. Growing demand, driven by population aging, rising chronic disease burden, and limited access to primary and urgent care, has pushed many EDs to operate at or beyond capacity, contributing to the persistent problem of ED crowding [1-5]. Conceptualized through the input-throughput-output model, crowding reflects a systems-level imbalance between patient demand and available hospital resources [2,4,6]. Input factors relate to arrival volume and acuity; throughput involves diagnostic and treatment processes within the ED; and output reflects the ability to admit or transfer patients, particularly when inpatient beds are unavailable [4-7]. Boarding of admitted patients remains one of the most significant contributors, reducing available ED beds and delaying care for incoming patients [5-8].

Various operational metrics, such as occupancy rate, waiting-room census, ambulance diversion hours, boarding duration, and ED length of stay (LOS), are used to quantify crowding [6-9]. Although these measures capture different aspects of the phenomenon, none fully encompass its complexity, and variations in measurement complicate comparisons across studies and settings [7-10]. Despite this heterogeneity, crowding consistently signals a mismatch between care demand and emergency or inpatient capacity [8-11].

ED crowding has been linked to delays in key clinical processes, including imaging for stroke, administration of analgesia, initiation of antibiotics, delivery of sepsis management, and time-sensitive trauma interventions [9-12]. Delays in triage, room placement, reassessment, and physician evaluation are similarly reported [10-12]. Such disruptions stem from resource constraints and workflow bottlenecks and are particularly consequential for conditions where timely care is essential [11,12].

Findings on the association between crowding and mortality remain mixed [7,9,12,13]. Some studies report increased mortality risk during crowded periods, while others find no independent association after adjusting for confounders [12,13]. These inconsistencies likely reflect differences in study design, crowding metrics, adjustment methods, and patient populations [9-13]. Nonetheless, the possibility that crowding contributes to preventable deaths highlights its importance as a patient-safety concern [11-13].

Crowding also generates broader system-level consequences, including prolonged hospital LOS, increased healthcare costs, higher rates of adverse events such as infections, and reduced access to emergency services through ambulance diversion [8-13]. These downstream effects reinforce a cycle of inefficiency that strains patients, clinicians, and health-system operations [10-13]. This systematic review aims to consolidate and critically evaluate current evidence on the relationship between ED crowding and mortality, delays in care, clinical outcomes, and system-level effects.

## Review

Methodology

Literature Search Strategy

This systematic review was conducted in accordance with the Preferred Reporting Items for Systematic Reviews and Meta-Analyses (PRISMA) 2020 guidelines [14]. Four electronic databases-PubMed, Scopus, Web of Science, and the Cochrane Library-were searched from inception through December 2025. The search combined controlled vocabulary and free-text terms related to ED crowding (e.g., "crowding", "overcrowding", "ambulance diversion", "boarding", "occupancy", "length of stay") and clinical outcomes (e.g., "mortality", "treatment delays", "process of care"). Boolean operators were used to maximize capture while maintaining conceptual specificity. Search strategies were adapted for each database. Only English-language, human, original quantitative studies were eligible. Reviews, commentaries, modeling-only studies, simulation research, and non-ED settings were excluded.

Eligibility Criteria

Inclusion criteria were defined using a Population-Exposure-Outcome framework. Studies were eligible if they assessed ED crowding using a measurable exposure, such as occupancy rate, ambulance diversion, ED census, waiting-room volume, boarding time, throughput measures, or LOS, and reported at least one patient-centered clinical outcome [15]. Eligible outcomes included mortality, treatment delays, process-of-care indicators, adverse clinical events, and healthcare utilization measures. Studies were excluded if they lacked a quantifiable crowding metric, did not report clinical outcomes, focused solely on staff or operational measures, were non-original research, used simulation data without patient outcomes, or were conducted outside the ED. No study-design restrictions were applied.

Study Selection

All retrieved records were imported into EndNote for automated and manual duplicate removal. Screening occurred in two stages by two independent reviewers. Titles and abstracts were first screened for relevance to ED crowding and patient outcomes. Full texts of potentially eligible studies were then reviewed in detail. Discrepancies were resolved through discussion, with a third reviewer available for adjudication. This process ensured reproducibility and minimized selection bias.

Data Extraction and Quality Appraisal

Two reviewers independently extracted data using a standardized form. Variables included study characteristics, sample size, population demographics, crowding metrics, exposure definitions, outcome measures, analytic methods, effect estimates, and reported limitations. Additional contextual information, such as triage systems and data sources, was also collected. Study quality was assessed using the National Institutes of Health (NIH) Quality Assessment Tool for Observational Cohort and Cross-Sectional Studies, evaluating clarity of research aims, population definition, reliability of exposure and outcome measures, control of confounding, and appropriateness of statistical analysis [16]. Quality ratings (good, fair, or poor) informed the interpretation of findings and the overall strength of evidence.

Results

Study Selection

**Figure 1:**
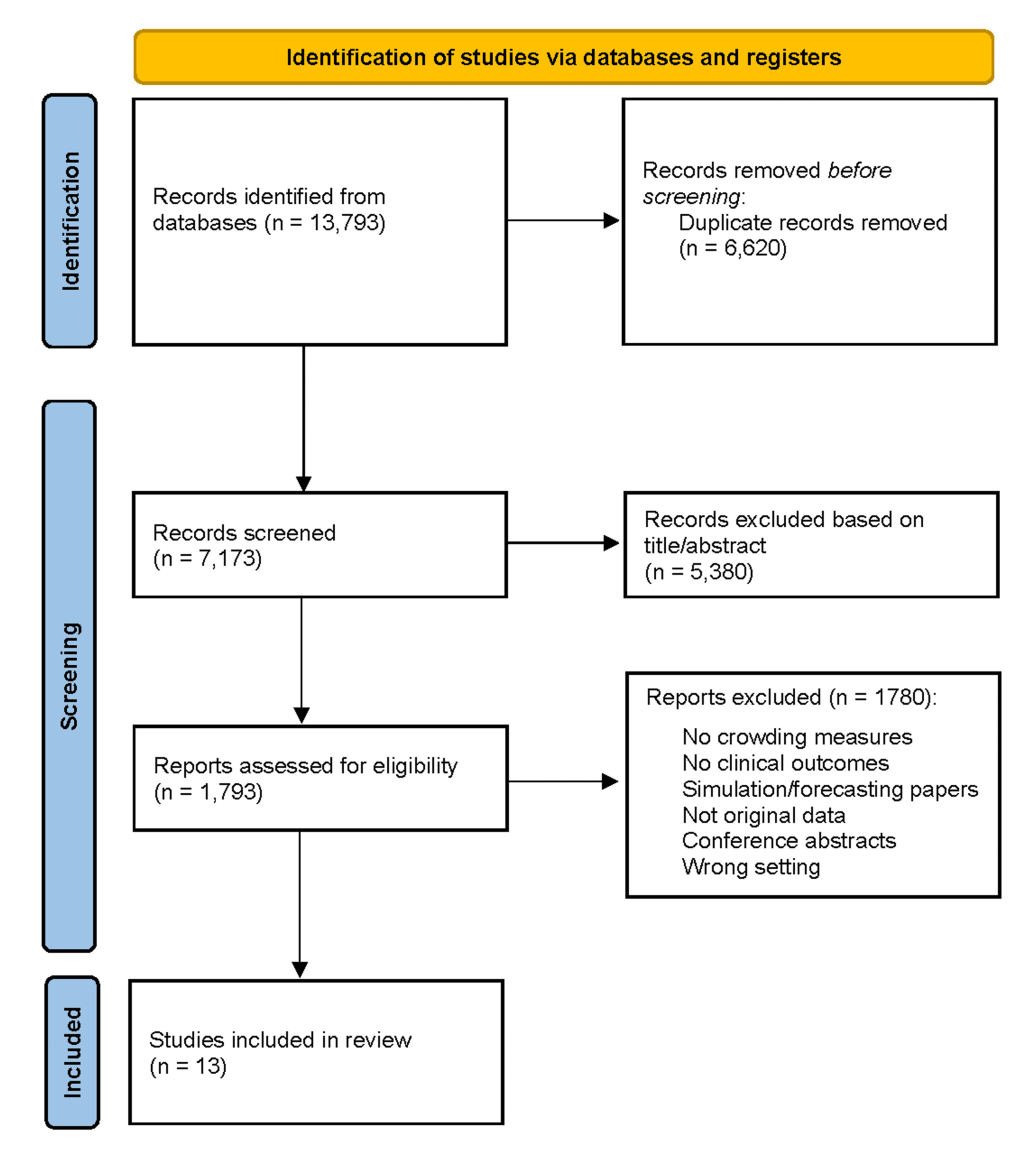
PRISMA flow diagram illustrating the study selection process PRISMA - Preferred Reporting Items for Systematic Reviews and Meta-Analyses

The database search identified 13,793 records from PubMed, Scopus, Web of Science, and the Cochrane Library. After the removal of 6620 duplicates, 7173 unique records underwent title and abstract screening. Most exclusions were due to the absence of an ED setting, lack of a measurable crowding exposure, simulation-only designs, non-clinical outcomes, or non-original research. A total of 1793 full-text articles were reviewed, and 1,780 were excluded for reasons including absence of quantifiable crowding metrics, lack of patient-centered outcomes, ineligible study design, non-ED environments, or incomplete reporting. Thirteen studies met all criteria and were included in the qualitative synthesis [1-13]. No meta-analysis was performed because of substantial variability in crowding definitions, exposure-time windows, patient populations, and outcome measures (Figure 1).

Study Characteristics

The 13 included studies were conducted across North America, Europe, and Asia and represented a spectrum of ED environments, including adult and pediatric academic centers, trauma systems, and large administrative datasets. Most were retrospective or prospective cohort studies, with sample sizes ranging from several hundred to millions of encounters. Populations included pediatric patients with abnormal vital signs, children with sickle cell crises, adults with transient ischemic attack or minor stroke, trauma patients with hemorrhagic shock, and general ED cohorts. Crowding metrics varied widely and encompassed arrival volume, waiting-room census, hourly or time-averaged occupancy, boarding duration, diversion hours, LOS, and other throughput indicators. Outcomes included delays in assessment or treatment, compliance with clinical pathways, resource utilization, adverse events, hospital LOS, and mortality. Statistical methods commonly included multivariable regression, Cox modeling, and hierarchical adjustment, with most studies controlling for age, sex, acuity, comorbidities, arrival mode, and severity scores. Across studies, a consistent pattern emerged linking ED crowding with impaired timeliness and quality of care, increased admissions, greater resource use, in some contexts-higher rates of complications or mortality (Table 1).

**Table 1:** Characteristics of studies included in the systematic review The table summarizes studies examining the impact of emergency department (ED) crowding on clinical processes and outcomes. Metrics include input (patients waiting), throughput (ED census, cumulative hours), and output (boarding). Outcomes assessed include mortality, delays in treatment, compliance with clinical bundles, hospital LOS, and adverse events. ED - emergency department; LOS - length of stay; VOC - vaso-occlusive crisis; ISS - Injury Severity Score; GCS - Glasgow Coma Scale; ESI - Emergency Severity Index; TTAS - Taiwan Triage and Acuity Scale; CTAS - Canadian Triage and Acuity Scale; CNS - Canadian Neurological Scale; DNAR - do-not-attempt-resuscitation; DCR - damage-control resuscitation; RBC - red blood cells; FFP - fresh frozen plasma; CVP - central venous pressure; MAP - mean arterial pressure; ScvO2 - central venous oxygen saturation; HR - hazard ratio; OR - odds ratio; aOR - adjusted odds ratio; SOFA - Sequential Organ Failure Assessment; NIHSS - National Institutes of Health Stroke Scale; EDOR - ED occupancy rate; AMA - against medical advice; LWBS - left without being seen; AUC - area under the curve; CMS - Centers for Medicare & Medicaid Services; EMR - electronic medical record; ESI - Emergency Severity Index

Study ID (Author)	Country & Setting	Study design & sample	Population (age, sex, criteria)	ED crowding definition & metrics	Exposure measures (waiting time, boarding, LOS)	Outcomes (mortality, delays, clinical outcomes)	Statistical findings (model, effect size, adjusted covariates)	Severity scores & data sources	Key findings
Sun et al. [1]	USA – 187 nonfederal California hospitals	Retrospective statewide cohort; 995,379 adult admissions	Adults ≥18 yrs; 47% male; all ED admissions except transfers, pediatric visits, hospitals without diversion	ED crowding via hospital-normalized ambulance diversion hours; high = top quartile diversion per facility	No direct LOS/boarding; diversion hours used as surrogate for ED saturation; median diversion = 7.1 hrs (IQR 3.6-11.2)	Primary: inpatient mortality. Secondary: hospital LOS, costs; 3-day mortality sensitivity	High crowding → ↑ mortality OR 1.05 (95% CI 1.02–1.08); ↑ LOS 0.8% (95% CI 0.5–1%); ↑ costs 1% (95% CI 0.7–2%). Sensitivity: three-day mortality ↑9%. Adjusted for demographics, 30 comorbidities, diagnosis, and hospital fixed effects	Severity approximated via Elixhauser comorbidities and CCS codes. Data from statewide discharge + EMS diversion logs	High ED crowding increases inpatient mortality, LOS, and costs. An estimated 300 excess deaths, 6200 excess hospital days, $17 excess costs/year. Demonstrates system-level quality/safety impact.
Reznek et al. [2]	USA – University of Massachusetts Medical Center, large academic ED	Retrospective cohort; 463 consecutive Code Stroke activations	Adults with suspected acute stroke ≤12 hrs from symptom onset. Median age 71; 59.6% female; 81% EMS arrivals; ESI 1–2 in 80%	ED occupancy rate (EDOR) = total ED patients ÷ licensed ED beds. Range 47–242% (median 122%). Also collected waiting room patients, admitted boarders	Exposure mismeasured as instantaneous EDOR at arrival. No direct LOS/boarding times; focus on real-time operational load	Primary: Door-to-Imaging Time (DIT) ≤25 min. Secondary: Door-to-Activation Time (DAT) ≤15 min	EDOR independently predicted failure to meet the DIT goal. Every 10% ↑ EDOR → odds of timely DIT ↓ by OR 0.83 (95% CI 0.75–0.91). Across 47–242% EDOR, adjusted OR ↓ to 0.025. Multivariate adjusted for 27 predictors; AUC 0.90–0.93	Stroke severity via NIHSS; ESI triage; glucose, vitals; mode of arrival; resuscitation room use; data from prospective stroke registry + EHR + operational logs	ED crowding markedly reduces timely brain imaging in acute stroke. Even after adjusting for patient and process factors, EDOR was a strong independent predictor. Delays increase sharply at higher crowding.
Shenoi et al. [3]	USA – Texas Children’s Hospital, urban tertiary pediatric ED	Cross-sectional; 232 encounters (161 unique sickle cell patients)	Children ≤20 yrs; median age 12; 94% Black; majority HbSS; included all VOC visits with pain; excluded fever-only, direct admissions, transfers, LWBS	ED census at arrival = total patients in ED (including fast-track). Quartiles used to categorize crowding; mean 57 patients (SD 24)	Median arrival→analgesic = 90 min (IQR 51–148). Delay mostly after triage, waiting for a room. ED LOS median 417 min	Primary: Analgesic delay (>60 min). Secondary: interval delays, association with pain score, acuity, arrival shift, Hgb type, disposition; no mortality	Logistic regression: ED census independently associated with delayed analgesia (OR 1.01 per patient increase, 95% CI 1.00–1.03). Severe pain reduced delay (OR 0.75). Younger age (<4 yrs OR 0.26). Discharge increased delay odds (OR 2.39). Good model fit	Triage acuity (5-level), 10-level pain score; data from EMSTAT ED tracking, nursing notes, and billing	ED crowding caused clinically significant delays in analgesic delivery for sickle cell VOC. Delay increased across ED census quartiles (p=0.0009), biphasic pattern with sharp delay beyond ~80 patients.
Wu et al. [4]	China – Zhejiang University Second Affiliated Hospital; urban Level I trauma center	Retrospective cohort; 3,037 trauma cases screened; 852 major trauma patients with hemorrhagic shock included	Adults >18 yrs; mean age 48.5; 72.1% male; ISS >16; hemorrhagic shock defined by hypotension + bleeding; excluded transfers, DNAR, refusal of therapy	Crowding is measured via the ED occupancy rate (patients in ED ÷ number of ED beds). Categorized into: Low (<90%), Medium (90–100%), High (>100%)	High crowding: delayed transfusion initiation (2.5 hrs vs. 1.0 low; p=0.01), reduced ED blood product availability, delayed procedures (e.g., DCS). ED LOS: 5.1 hrs high vs. 3.2 low	Delays in DCR bundle: transfusion initiation, surgery timing, RBC/FFP administration, urinary output monitoring. Secondary: traumatic coagulopathy, early lactate clearance, ICU LOS, 30-day mortality	Multivariate analysis: high crowding increased odds of delayed transfusion (OR 2.63), fewer RBC units (OR 0.62), fewer FFP units (OR 0.69), delayed DCS (OR 1.85), and more traumatic coagulopathy (OR 1.66). No association with lactate clearance, ICU LOS, or 30-day mortality	ISS and GCS are used for severity scoring. Data from the trauma registry, the ED triage system, and the hospital medical records	ED crowding worsened DCR performance, delaying transfusion/surgery and reducing blood product delivery. Increased traumatic coagulopathy and ED LOS, but no increase in 30-day mortality.
Verelst et al. [5]	Belgium – University Hospitals Leuven, a large tertiary academic center	Prospective observational cohort, 2 years; 108,229 adult ED visits, 32,866 hospital admissions	Adults ≥18 yrs; median age ED-only ~32, admitted ~65–68; 51–54% male; triaged via ESI	ED occupancy rate, updated every 10 min. Average ED occupancy measured from 4 hrs before to 4 hrs after arrival; Q4 highest quartile, Q1 reference	Crowding quartile Q4: median 58 patients for 40 licensed bays. Boarding is not separately measured; total ED LOS is tracked	Primary: in-hospital mortality ≤10 days. Secondary: 5 AHRQ-defined adverse events, hospital LOS, ED LOS	Adjusted for illness	Administrative data; no severity score; diagnosis groups via CCS codes	ED crowding (ambulance diversion) is not associated with seven-day bounceback admissions; bounceback is driven more by patient factors than ED crowding.
Derose et al. [6]	USA – Kaiser Permanente Southern California; 13 EDs	Retrospective cohort; 136,740 adult admissions	Adults ≥18 yrs; mean age ~61; 47% male; admitted through ED; excluded observation, hospice, transfers, nonmembers	Multiple ED crowding metrics: (1) transit times (waiting, evaluation, boarding, ED LOS); (2) system crowding (occupancy at entry, time-averaged occupancy, ED LOS at entry, boarding time)	Median values: waiting 0.56h; eval/treatment 4.3h; boarding 2.5h; ED LOS 7.34h. Boarding varied up to 14+ hrs; occupancy 0.7–0.9 persons/bed	Primary: inpatient mortality. Secondary: admission hospital LOS; relationship to CMS ED-1b/ED-2b metrics	After adjustment, no ED crowding metric predicted inpatient mortality. Boarding time associated with longer admission LOS: first 14 hrs of boarding added ~6 hrs of hospital LOS.	Triage categories 1–5. Data from the ED information system with real-time timestamps, bed occupancy logs	ED occupancy inversely correlated with 4-h performance. Stable up to ~70%, then linear deterioration. Peak occupancy >21 beds or daily >65% predicts failure.
Hsia et al. [7]	USA – 202 California acute-care hospitals	Retrospective statewide cohort; 3,368,527 adult ED discharges	Adults ≥18 yrs; excluded transfers, hospice, deaths in ED, missing identifiers, pediatric visits	Crowding via hospital-specific daily ambulance diversion hours; high if top quartile per hospital	High crowding days accounted for 596,471 visits (17.7%). No LOS or boarding data; exposure = diversion hours/day	Primary: seven-day bounceback admission. Secondary: three-day bounceback sensitivity	Hierarchical logistic regression: no association between crowding and bounceback (OR 1.01, 95% CI 0.99–1.02). Covariates: age, AMA/elope, Medicare/Medicaid, hospital type	SOFA score at diagnosis. Data from hospital EHR + sepsis registry	ED crowding reduced sepsis bundle compliance (32% → 16%), delayed all bundle elements, and increased ED LOS. Mortality is slightly higher (18.4%) but not statistically significant.
Higginson et al. [8]	UK – Derriford Hospital, large tertiary ED & trauma center	Retrospective observational; 43,799 ED attendances	Mean age 56 yrs; 49% male; included all adult ED patients; 56% admitted	ED occupancy rate measured three ways: (1) minutes/day spent at 70–100% occupancy, (2) peak occupancy of majors/resus beds, (3) % of total possible daily bed-minute occupancy	No direct LOS/boarding; crowding exposure quantified via occupancy burden, peak bed saturation, total daily occupancy	Primary: performance against four-hour ED standard. Secondary: threshold identification for crowding-related deterioration. No mortality/clinical harm outcomes	Strong inverse correlation between occupancy and 4-h performance: 80% (r=–0.33), 90% (r=–0.52), 100% (r=–0.67), p<0.001. Peak occupancy >21 beds predicted failure of four-hour target	No formal severity score; acuity inferred from mental status & critical care needs. Data from the NEDIS national registry with real-time ED logs	A large national study showed that ED crowding increased pediatric inpatient mortality by 26% and prolonged boarding times.
Shin et al. [9]	South Korea – Samsung Medical Center, Seoul; urban tertiary referral ED	Retrospective observational cohort using sepsis registry; n = 770 severe sepsis/septic shock patients	Adults ≥18 yrs; median age 65; 56.8% male; included severe sepsis or septic shock with lactate ≥4 mmol/L; excluded terminal malignancy, DNAR, treatment refusal	ED occupancy rate (patients in ED ÷ ED beds), validated measure. Categorized: low (≤115%), intermediate (116–149%), high (≥150%). Calculated at triage	Time to bundle elements significantly longer in high crowding: antibiotics (2.8 hrs vs 2.4 low), lactate, cultures, CVP, MAP, ScvO2 targets. ED LOS increased: 24 hrs high vs 16 hrs low	Primary: overall compliance with full resuscitation bundle. Secondary: completion of each bundle element, number of interventions completed, ED LOS, ICU LOS, in-hospital LOS, in-hospital mortality	High crowding ↓ odds of bundle compliance (aOR 0.44, 95% CI 0.26–0.76, p=0.003). Each +10% occupancy ↓ compliance (OR 0.90, 95% CI 0.84–0.96). High crowding ↓ , early antibiotics (aOR 0.57), ↓ and ScvO2 achievement (aOR 0.42). Adjusted for age, sex, SOFA, lactate, arrival time, and physician experience	Severity: Canadian Neurological Scale (CNS). Triaged using CTAS. Data from the Ontario Stroke Registry linked with administrative databases	ED crowding reduced the likelihood of discharge for TIA/minor stroke. Physicians admitted more during crowded shifts; crowding did not increase 7- or 30-day readmission, stroke, or death among discharged patients.
Cha et al. [10]	South Korea – 34 mixed adult–pediatric EDs using the national NEDIS database	Cross-sectional multicenter observational; 125,031 admitted pediatric patients	Pediatric patients <15 yrs; mean age 3.84 yrs; 59.3% male; included all admitted cases; exclusions: pediatric-only EDs, hospice hospitals, EDs with no pediatric deaths	Crowding is defined by ED patient volume exceeding the monthly highest quartile during an 8-hour shift; EDs are preselected as “potentially crowded” if the mean boarding >6 hrs	Boarding times longer in crowded vs non-crowded: general ward (10.23 vs 8.67 hrs), OR transfers (8.22 vs 7.46 hrs). ICU boarding similar	Primary: 30-day hospital mortality. Secondary: factors associated with mortality (injury, surgery, mental status, critical care)	Adjusted Cox model: crowded EDs ↑ mortality risk (HR 1.260, 95% CI 1.019–1.558, p=0.03). Significant covariates: age 2–5 yrs (protective), female sex (HR 1.30), low consciousness (HR 3.4–7.1), critical illness (HR 23.1). Controlled for insurance, surgery, injury, critical care, and day of week	Used ESI triage; data from EmSTAT EHR: timestamps, vital signs, staffing, ED flow metrics	ED crowding significantly delayed reassessment of critically abnormal vital signs in children. Output crowding (boarding) had the strongest impact, reducing reassessment rates by up to 31%.
Ben-Yakov et al. [11]	Canada – 12 regional ED stroke centers in Ontario; tertiary hospitals	Retrospective cohort using prospectively collected registry data; n=9759 patients (4607 TIA, 5152 minor stroke)	Mean age 70.8 ± 13.4; 52.9% male; CTAS 1–5; included adults with TIA or minor ischemic stroke (CNS ≥ 9). Excluded moderate/severe stroke, transfers, <18 years	Crowding is measured as acuity-standardized mean ED LOS for patients of similar CTAS level seen on the same shift (8-hour blocks). Crowding categorized: 0–<4 hrs, 4–<5, 5–<6, 6–<7, ≥7 hrs	Crowded shift is defined as mean ED LOS >4 hrs. TIA/stroke patients experienced longer LOS and higher resource utilization. Highest crowding group: ≥7 hrs	Primary outcome: disposition (admit vs discharge). Secondary: 7- and 30-day death, stroke, readmission	Hierarchical logistic regression: higher ED LOS = decreased odds of discharge. Patients are more likely to be admitted when the ED is more crowded. AUC = 0.83. Adjusted for age, sex, CTAS, comorbidities, investigations, ECG/CT use, neurology consult, arrival mode, income, time/day, hand ospital characteristics. No association between crowding and adverse events post-discharge	Severity is measured by the TTAS triage levels. Data from ED administrative databases	Higher ED occupancy worsened clinical efficiency, increased hospital admissions, mildly increased CT/lab use, no impact on ED mortality.
Depinet et al. [12]	USA – Urban tertiary pediatric ED (Cincinnati Children’s)	Retrospective cross-sectional; 9976 encounters with critically abnormal triage vital signs	Age 0–21 years; 45% female; included children with triage VS outside age-adjusted norms; excluded trauma bay cases, transfers, non-triage abnormal VS	Crowding measured with input (patients waiting), throughput (hourly ED census, cumulative ED hours), output (number boarding, cumulative boarding hours); metrics captured hourly	Mean reassessment time: 84.7 ± 57.1 min; ED census mean 53.8; patients waiting for room mean 24.7; boarding mean 4.4	Primary outcome: delay in vital-sign reassessment; no mortality outcomes; clinical implication = delayed monitoring of unstable children	Cox proportional hazards model: reassessment rate decreased by 31% per +10 patients awaiting admission (HR=0.69), 10% per +10 lobby patients (HR=0.94), 6% per +10 ED census (HR~0.94–0.97), 7% per +100 cumulative ED hours (HR=0.93), 2% per +4 boarding hours (HR=0.98). Adjusted for age, race, shift, acuity, nurse staffing, and admission	ESI triage levels, vitals, comorbidities via Elixhauser index, administrative + EHR timestamps	Crowding did not increase inpatient mortality once adjusted. Boarding time robustly associated with prolonged admission LOS; most actionable crowding metric.
Chiu et al. [13]	Taiwan – Two large tertiary EDs	Retrospective one-year cohort; 70,222 adult non-trauma ED visits	Adults ≥18 yrs; mean age ~57; ~50% male; triaged using Taiwan TTAS (five-level system)	ED occupancy status = number of patients physically in ED at visit time; grouped into quartiles: <24, 24–39, 39–62, >62	Higher quartiles associated with longer decision-making time (+0.3 hr) and ED LOS (+1.1 hr). Boarding to observation/ward/ICU increased	Primary: decision-making time, ED LOS. Secondary: admissions to observation, ward, ICU; ED mortality; use of CT & labs	Multinomial & binomial logistic regression: compared to lowest crowding: ↑ ED observation (aOR up to 5.2), ↑ ward admission (aOR up to 2.5), ↑ ICU admission (aOR up to 2.7). CT use ↑ only in 4th quartile (aOR 1.1). Adjusted for age, sex, acuity, hospital, diagnosis, day & month		

Quality Assessment

Methodological quality was generally acceptable, with most studies clearly defining their populations, measuring exposures before outcomes, and achieving high completeness of data. Exposure and outcome reliability were strong in studies using continuous occupancy tracking or administrative datasets, with several achieving excellent reliability ratings [2,6,8]. Loss to follow-up was minimal. Statistical modeling typically adjusted for major confounders, supporting internal validity. Limitations included the absence of sample-size justification, inconsistent use of repeated crowding measurements, variable reliability for indirect exposure measures such as diversion hours, and lack of assessor blinding, although this was often not applicable to retrospective designs. Confounding control ranged from moderate to strong, resulting in overall quality ratings between fair and good (Table 2).

**Table 2:** Quality assessment of included studies This table summarizes the methodological quality of included studies assessing emergency department (ED) crowding and its impact on clinical outcomes. Columns indicate whether the research question was clearly stated, study population was clearly defined, participation rate was ≥50%, participants were recruited from the same population and time period, sample size was justified, exposure was measured before the outcome, exposure was assessed more than once, exposure was measured reliably, outcome was clearly defined, outcome was measured reliably, outcome assessors were blinded, loss to follow-up was <20%, confounding factors were measured and adjusted, and statistical methods were appropriate. Overall quality ratings (Good, Fair, Fair–Good, Moderate) reflect the cumulative assessment of these criteria. ED - emergency department; NA - not applicable

Study ID	Research question clearly stated	Study population clearly defined	Participation rate ≥50%	Recruited from same population & time period	Sample size justified	Exposure measured before outcome	Exposure assessed more than once	Exposure measured reliably	Outcome clearly defined	Outcome measured reliably	Outcome assessors blinded	Loss to follow-up <20%	Confounding measured & adjusted	Statistical methods appropriate	Overall quality
Sun et al. [1]	Yes	Yes	Yes	Yes	No	Yes	NA	Moderate	Yes	Yes	NA	Yes	Excellent	Yes	Good
Reznek et al. [2]	Yes	Yes	Yes	Yes	No	Yes	No	Excellent	Yes	Yes	NA	NA	Yes	Yes	Good
Shenoi et al. [3]	Yes	Yes	Yes	Yes	No	Yes	No	Moderate	Yes	Fair	NA	NA	Moderate	Yes	Fair–Good
Wu et al. [4]	Yes	Yes	Yes	Yes	No	Yes	No	Yes	Yes	Yes	NA	NA	Moderate	Yes	Fair–Good
Verelst et al. [5]	Yes	Yes	Yes	Yes	No	Yes	No	Good	Yes	Moderate	NA	NA	Moderate	Yes	Fair–Good
Derose et al. [6]	Yes	Yes	Yes	Yes	No	Yes	Yes	Excellent	Yes	Yes	NA	NA	Yes	Yes	Good
Hsia et al. [7]	Yes	Yes	Yes	Yes	No	Yes	NA	Moderate	Yes	Yes	NA	Yes	Moderate	Yes	Fair
Higginson et al. [8]	Yes	Yes	Yes	Yes	No	Yes	Yes	Excellent	Yes	Yes	NA	NA	No	Yes	Fair
Shin et al. [9]	Yes	Yes	Yes	Yes	No	Yes	Yes	Yes	Yes	Fair	NA	NA	Yes	Yes	Good
Cha et al. [10]	Yes	Yes	Yes	Yes	No	Yes	No	Moderate	Yes	Yes	NA	Yes	Fair	Yes	Fair
Ben-Yakov et al. [11]	Yes	Yes	Yes	Yes	No	Yes	Yes	Yes	Yes	Yes	NA	Yes	Yes	Yes	Good
Depinet et al. [12]	Yes	Yes	Yes	Yes	No	Yes	Yes	Yes	Yes	Yes	NA	NA	Yes	Yes	Good
Chiu et al. [13]	Yes	Yes	Yes	Yes	No	Yes	No	Yes	Yes	Yes	NA	NA	Yes	Yes	Good

Synthesis of Findings

Across all studies, ED crowding was strongly associated with delays in time-sensitive clinical processes. Pediatric studies showed prolonged reassessment of abnormal vital signs and delayed analgesia administration correlated with higher ED census and occupancy levels [3]. In adult populations, higher occupancy reduced compliance with guideline-recommended door-to-imaging targets for stroke evaluation and prolonged decision-making time and ED LOS [2,13]. Trauma studies reported slower initiation of transfusion and reduced efficiency of damage-control resuscitation during crowded conditions [4].

Crowding also disrupted clinical processes and quality-of-care indicators. Patients with transient ischemic attack or minor stroke were less likely to be discharged during crowded periods, suggesting more conservative disposition patterns. Increased admission rates, observation-unit use, and intensive care unit (ICU) transfers were observed with rising occupancy [13]. In sepsis care, bundle compliance declined substantially as crowding increased, driven by delayed antibiotics, lactate measurement, and hemodynamic reassessment [9]. System-level performance metrics such as four-hour disposition targets deteriorated sharply beyond critical occupancy thresholds [8].

Effects on adverse outcomes varied by population. Pediatric inpatient mortality increased under crowded conditions in unadjusted analyses but lost significance after adjustment for severity, although boarding times remained longer and potentially hazardous [10]. In adult cohorts, crowding was not consistently associated with short-term mortality after controlling for acuity, but increased rates of complications, such as hospital-acquired pneumonia and prolonged hospital LOS, were observed [5]. Trauma patients showed no mortality association but demonstrated higher rates of traumatic coagulopathy during crowded periods [4]. Large administrative data demonstrated that ambulance diversion, a surrogate for severe crowding, was associated with increased inpatient mortality, longer LOS, and higher costs at the population level [1]. Short-term return admissions showed no relationship to crowding, suggesting limited sensitivity of bounceback metrics [7].

Crowding also had measurable financial and utilization impacts. Increased boarding time and prolonged ED LOS translated into longer inpatient stays and higher system-wide costs [1,5,6]. Admission rates rose across all levels of care, and inpatient bed inefficiency propagated downstream delays and resource strain [13].

Limitations

This review has several limitations. Most included studies were observational, which restricts causal inference and leaves residual confounding despite multivariable adjustment. Considerable heterogeneity existed in crowding definitions, measurement tools, and exposure timeframes, limiting comparability across studies and precluding meta-analysis. Differences in patient populations, illness severity, outcome definitions, and modeling approaches may also explain variation in reported effects, particularly for mortality. Many studies originated from single centers or high-resource healthcare systems, reducing generalizability to low- and middle-income settings. Finally, publication bias may favor studies reporting adverse effects of crowding, potentially inflating the perceived magnitude of its impact.

## Conclusions

This systematic review demonstrates that emergency department crowding has consistently negative effects on the timeliness and quality of care, operational performance, and downstream clinical outcomes. Crowding contributes to delays in essential assessments and treatments, prolongs ED and inpatient LOS, increases the risk of complications, and disrupts workflow efficiency. Although findings on mortality remain variable, the overall evidence indicates that crowding represents a significant threat to patient safety and health-system functioning. Addressing this challenge requires coordinated, system-wide approaches that improve patient flow, reduce boarding, and expand bed capacity. Effective and sustained interventions are essential to mitigate the clinical and operational burdens of ED crowding and to ensure timely, high-quality emergency care.
